# Supplementary material for: HIV serologically indeterminate individuals: Future HIV status and risk factors
Source: PLoS One. 2020 Aug 26;15(8):e0237633. doi: 10.1371/journal.pone.0237633 (PMC7449388; doi:10.1371/journal.pone.0237633)
Supplement: S1 Fig — (DOCX) [file pone.0237633.s001.docx]

S1 Fig. HIV EIA Parallel Testing Algorithm
