# Supplementary material for: HIV serologically indeterminate individuals: Future HIV status and risk factors
Source: PLoS One. 2020 Aug 26;15(8):e0237633. doi: 10.1371/journal.pone.0237633 (PMC7449388; doi:10.1371/journal.pone.0237633)

Supplemental figure 3. Future HIV EIA results for individuals with HIV negative results at their first visit

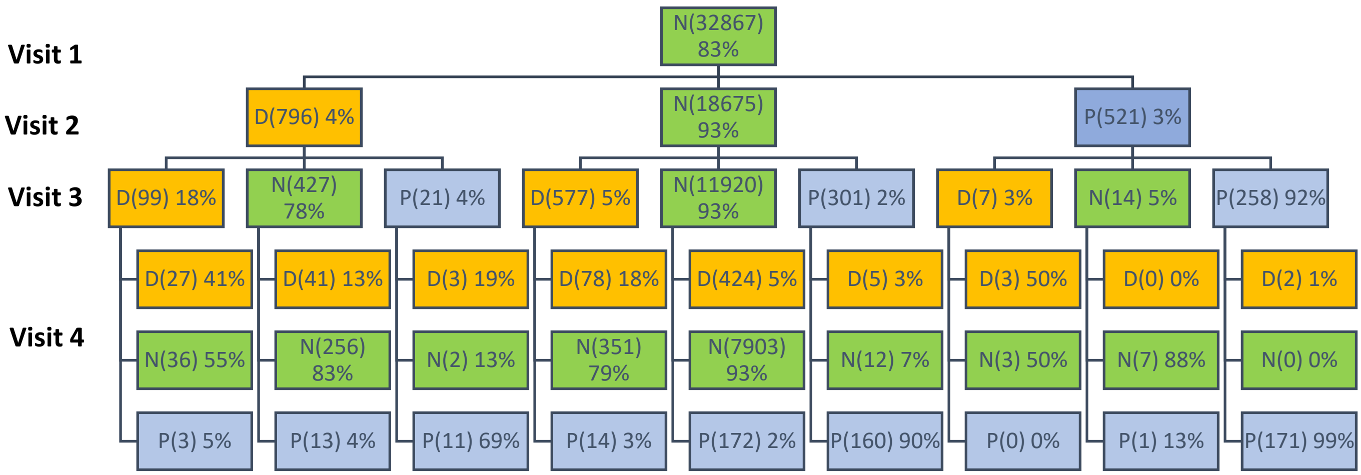

Supplement: S3 Fig — (PDF) [file pone.0237633.s003.pdf]
