# Supplementary material for: HIV serologically indeterminate individuals: Future HIV status and risk factors
Source: PLoS One. 2020 Aug 26;15(8):e0237633. doi: 10.1371/journal.pone.0237633 (PMC7449388; doi:10.1371/journal.pone.0237633)
Supplement: S5 Fig — (PDF) [file pone.0237633.s005.pdf]

Supplemental figure 5. Prevalence of HIV EIA Indeterminate results for RCCS participants in Rakai, Uganda (1994-2009).

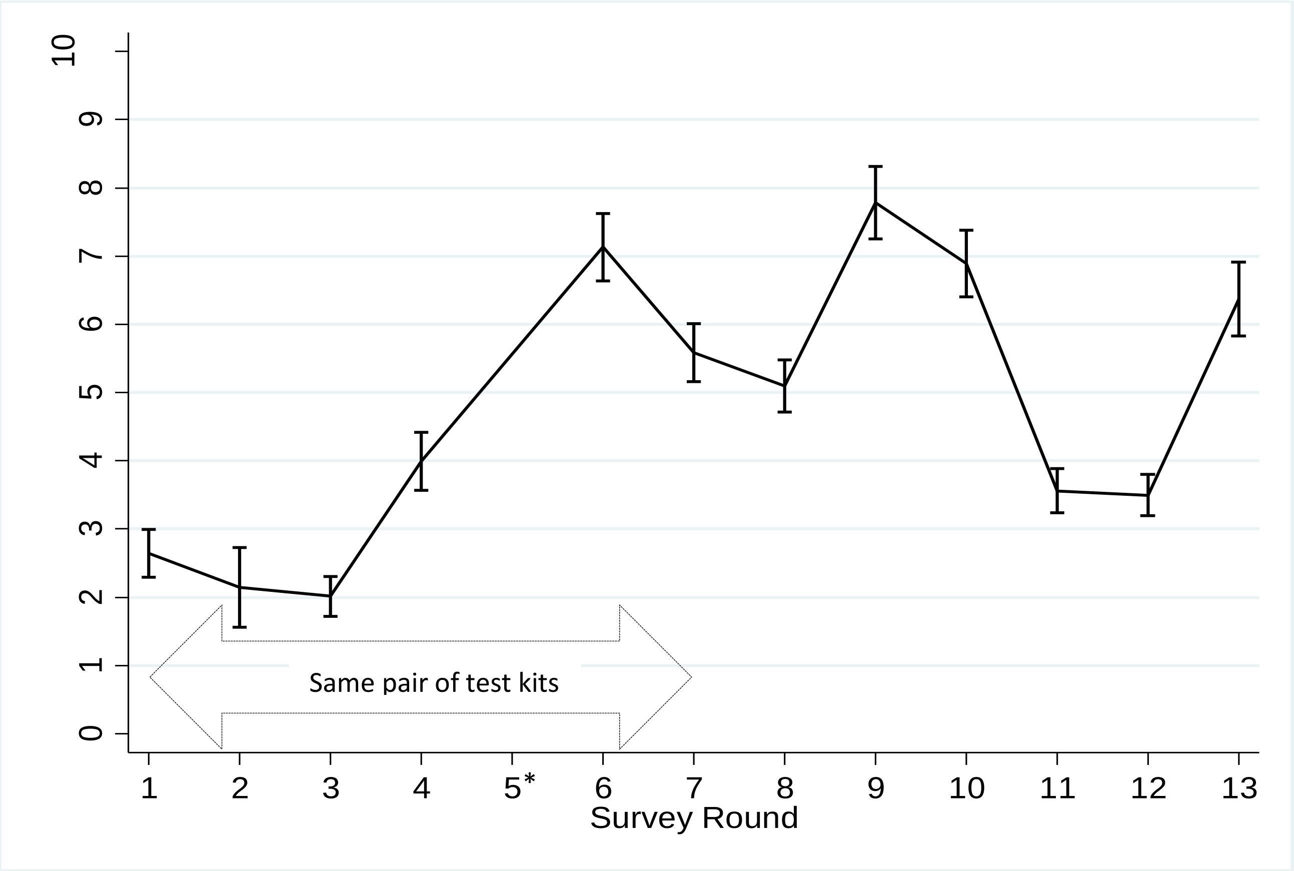

*Survey round 5 was excluded from this analysis due to sample size restrictions
