# Supplementary material for: HIV serologically indeterminate individuals: Future HIV status and risk factors
Source: PLoS One. 2020 Aug 26;15(8):e0237633. doi: 10.1371/journal.pone.0237633 (PMC7449388; doi:10.1371/journal.pone.0237633)
Supplement: S1 Table — (DOCX) [file pone.0237633.s006.docx]

| S1 Table. Factors associated with having two or more HIV serologically indeterminate results among 20000 (87945 person-visits) RCCS participants in Rakai, Uganda (1994-2009). | | | | |
| --- | --- | --- | --- | --- |
| Risk Factors | **Observations (%)** | **EIA_i_ prevalence** | **UnadjPR (95% CI)** | **AdjPR (95% CI)*** |
| Marital Status |  |  |  |  |
| Married | 57788(66) | 4201/57788= 7.3% | 1.00 | 1.00 |
| Not married | 30157(34) | 1510/30157= 5.0% | 0.69(0.61,0.78) | 0.85(0.73,0.98) |
| Gender |  |  |  |  |
| Male | 38520(44) | 2822/38520= 7.3% | 1.00 | 1.00 |
| Female | 49425(56) | 2890/49425= 5.9% | 0.80(0.70,0.91) | 0.73(0.63,0.85) |
| Education |  |  |  |  |
| No education | 6287(7) | 459/6287= 7.3% | 1.00 | 1.00 |
| Primary | 58004(66) | 4088/58004= 7.1% | 0.97(0.75,1.25) | 1.03(0.80,1.33) |
| Secondary | 20760(24) | 993/20760= 4.8% | 0.66(0.49,0.88) | 0.77(0.57,1.05) |
| Tertiary | 2894(3) | 172/2894= 5.9% | 0.81(0.50,1.32) | 0.82(0.49,1.38) |
| Resident |  |  |  |  |
| Rural | 54446(62) | 3875/54446= 7.1% | 1.00 | 1.00 |
| Urban/trading | 33499(38) | 1837/33499= 5.5% | 0.77(0.67,0.89) | 0.80(0.68,0.96) |
| Religion |  |  |  |  |
| None | 411(0.5) | 38/411= 9.3% | 1.00 | 1.00 |
| Other | 512(0.6) | 10/512= 2.0% | 0.21(0.05,0.96) | 0.25(0.06,1.10) |
| Catholic | 52760(60) | 3462/52760= 6.6% | 0.71(0.28,1.80) | 0.81(0.33,1.97) |
| Muslim | 13462(15) | 854/13462= 6.3% | 0.69(0.27,1.77) | 0.83(0.34,2.03) |
| Protestant | 19002(22) | 1285/19002= 6.8% | 0.73(0.29,1.87) | 0.82(0.34,2.01) |
| Saved/Pentecostal | 1798(2) | 63/1798= 3.5% | 0.38(0.13,1.10) | 0.46(0.17,1.28) |
| No. sex partners |  |  |  |  |
| 0 | 13262(15) | 666/13262= 5.0% | 1.00 | 1.00 |
| 1 | 58617(67) | 3918/58617= 6.7 % | 1.33(1.15,1.54) | 1.00(0.86,1.17) |
| 2 | 11105(13) | 792/11105= 7.1% | 1.42(1.18,1.70) | 0.93(0.77,1.12) |
| 3 | 3215(4) | 219/3215= 6.8% | 1.36(1.07,1.72) | 0.89(0.69,1.14) |
| 4 | 792(1) | 43/792= 5.4% | 1.08(0.75,1.56) | 0.75(0.51,1.09) |
| 5+ | 954(1) | 74/954= 7.8% | 1.54(1.07,2.24) | 1.10(0.75,1.60) |
| Occupation |  |  |  |  |
| Agriculture | 45752(52) | 3302/45752= 7.2% | 1.00 | 1.00 |
| Housework | 3436(4) | 200/3436= 5.8% | 0.81(0.55,1.18) | 1.14(0.78,1.67) |
| Bar/Brewer/saloon | 930(1) | 83/930= 8.9% | 1.24(0.71,2.15) | 1.29(0.74,2.22) |
| Government/salaried | 5802(7) | 343/5802= 5.9% | 0.82(0.61,1.09) | 0.98(0.70,1.37) |
| Other | 11248(13) | 682/11248= 6.1% | 0.84(0.68,1.04) | 0.89(0.72,1.11) |
| Student | 11028(13) | 438/11028= 4.0% | 0.55(0.43,0.70) | 0.81(0.61,1.09) |
| Shopkeeper/Trading | 9429(11) | 651/9429= 6.9% | 0.96(0.77,1.20) | 0.94(0.75,1.19) |
| Truck driver | 320(0.4) | 13/320= 4.1% | 0.56(0.12,2.61) | 0.62(0.13,2.83) |
| Age |  |  |  |  |
| 15-19 | 14643(17) | 583/14643= 4.0% | 1.00 | 1.00 |
| 20-24 | 19572(22) | 1086/19572= 5.6% | 1.39(1.24,1.56) | 1.25(1.10,1.42) |
| 25-29 | 18151(21) | 1297/18151= 7.2% | 1.79(1.56,2.07) | 1.54(1.30,1.82) |
| 30-34 | 13347(15) | 1038/13347= 7.8% | 1.95(1.66,1.29) | 1.62(1.34,1.97) |
| 35-39 | 9629(11) | 783/9629= 8.1% | 2.04 (1.72,2.43) | 1.67(1.36,2.05) |
| 40-49 | 12603(14) | 925/12603= 7.3% | 1.84(1.52,2.23) | 1.53(1.23,1.91) |
| Malaria |  |  |  |  |
| Yes | 2398(3) | 106/2398= 4.4% | 1.00 | 1.00 |
| No | 85547(97) | 5606/85547= 6.6% | 1.48(1.19,1.84) | 1.32(1.07,1.64) |

*model also adjusted for visits and region of residence, EIA_i_ = Enzyme-linked Immunoassay indeterminate
