# Supplementary material for: HIV serologically indeterminate individuals: Future HIV status and risk factors
Source: PLoS One. 2020 Aug 26;15(8):e0237633. doi: 10.1371/journal.pone.0237633 (PMC7449388; doi:10.1371/journal.pone.0237633)
Supplement: S2 Table — (DOCX) [file pone.0237633.s007.docx]

S2 Table. The prevalence of transitioning from HIV (NN), (PP) or (DD) to HIV negative (N), positive (P) or indeterminate (D) in subsequent follow-up serological test.

|  | # of Obs. (%) | Subsequent Follow-up EIA Result | | |
| --- | --- | --- | --- | --- |
|  |  | HIV+ (P) | HIV- (N) | Indeterminate (D) |
| Lag result EIA |  |  |  |  |
| NN | 41562(87) | 868/41562= 2% | 38770/41562= 93% | 1924/41562= 5% |
| DD | 628(1) | 31/628= 5% | 311/628= 50% | 286/628= 4% |
| PP | 5362(11) | 5321/5362= 99% | 12/5362= 0.2% | 29/5362= 0.5% |

EIA = Enzyme-linked Immunoassay
