# Supplementary material for: HIV serologically indeterminate individuals: Future HIV status and risk factors
Source: PLoS One. 2020 Aug 26;15(8):e0237633. doi: 10.1371/journal.pone.0237633 (PMC7449388; doi:10.1371/journal.pone.0237633)
Supplement: S3 Table — (DOCX) [file pone.0237633.s008.docx]

S3 Table. Test kit used for each survey round and the percentage of samples tested by each test kit

| Survey Round | Testkits (% of sample tested) |
| --- | --- |
| 1-8 | C- CAMBRIDGE (100),  O- ORGANON Teknika (100) |
| 9 | C- CAMBRIDGE (75.5),  O- ORGANON (99.8),  M- MUREX(0.2),  W- WELCOZYME(24.4),  U- UNIFORM(0.2) |
| 10 | O- ORGANON(63.6),  M- MUREX(16.0),  W- WELCOZYME(84.0),  U- UNIFORM(36.4) |
| 11 | M- MUREX(100),  U- UNIFORM(99.98),  O- ORGANON(0.02) |
| 12 | M- MUREX(100),  U- UNIFORM(100) |
| 13 | M- MUREX(100),  U-UNIFORM(100) |
|  |  |
| Sensitivit and specificity of each test kit as reported by the manufacturer | |
| Test kit | **Sensitivity/specificity** |
| BioRad GS HIV -1/HIV-2 plus O | 100/99.89 |
| Cambridge | 100/100 |
| Murex HIV 1.2 | 100/99.91 |
| Organon Teknika | 100/99.92 |
| Vironostika HIV Uni-form 11 plus O | 100/100 |
| Welcozyme | 100/99 |
