# Supplementary material for: HIV serologically indeterminate individuals: Future HIV status and risk factors
Source: PLoS One. 2020 Aug 26;15(8):e0237633. doi: 10.1371/journal.pone.0237633 (PMC7449388; doi:10.1371/journal.pone.0237633)
Supplement: S4 Table — (DOCX) [file pone.0237633.s009.docx]

| S4 Table. Factors associated with HIV serologically indeterminate results among 26,375 (62,148 person-visits) RCCS participants in Rakai, Uganda (1994-2002). | | | | |
| --- | --- | --- | --- | --- |
| Factors | **Observations (%)** | **EIA Indeterminate_i_ prevalence** | **UnadjPR (95% CI)** | **AdjPR (95% CI)*** |
| Marital Status |  |  |  |  |
| Married | 38558(62) | 959/23590 = 4.1% | 1.00 | 1.00 |
| Not married | 23590(38) | 1840/38558= 4.8% | 0.86(0.79,0.94) | 0.89(0.80,0.99) |
| Gender |  |  |  |  |
| Male | 26874(43) | 1352/26874 = 5.0% | 1.00 | 1.00 |
| Female | 35274(57) | 1447/35274= 4.1% | 0.81(0.74,0.88) | 0.78(0.71,0.86) |
| Education |  |  |  |  |
| No education | 5078(8) | 260/5078= 5.1% | 1.00 | 1.00 |
| Primary | 41016(66) | 1870/41016= 4.6% | 0.90(0.77,1.04) | 0.83(0.71,0.97) |
| Secondary | 13618(22) | 574/13618= 4.2% | 0.82(0.69,0.97) | 0.73(0.61,0.87) |
| Tertiary | 2436(4) | 95/2436= 3.9% | 0.75(0.57,98) | 0.63(0.47,0.85) |
| Resident |  |  |  |  |
| Rural | 38487(62) | 1739/38487= 4.5% | 1.00 | 1.00 |
| Urban/trading | 23661(38) | 1060/23661= 4.5% | 0.97(0.89,1.05) | 0.97(0.87,1.08) |
| No. sex partners |  |  |  |  |
| 0 | 10587(17) | 406/10587= 3.8% | 1.00 | 1.00 |
| 1 | 40146(65) | 1799/40156= 4.5% | 1.17(1.05,1.31) | 1.06(0.93,1.21) |
| 2 | 7730(12) | 390/7730= 5.1% | 1.32(1.14,1.52) | 1.08(0.93,1.27) |
| 3 | 2289(4) | 116/2289= 5.1% | 1.34(1.09,1.64) | 1.07(0.86,1.33) |
| 4 | 585(1) | 30/585= 5.1% | 1.42(1.01,1.99) | 1.11 (0.79,1.58) |
| 5+ | 801(1) | 58/801= 7.2% | 1.84(1.39,2.43) | 1.39(1.04,1.85) |
| Age |  |  |  |  |
| 15-19 | 12768(21) | 538/12768= 4.2% | 1.00 | 1.00 |
| 20-24 | 14248(23) | 648/14248= 4.6% | 1.10(0.98,1.23) | 0.95(0.84,1.07) |
| 25-29 | 12129(20) | 557/12129= 4.6% | 1.09(0.96,1.24) | 0.88(0.77,1.02) |
| 30-34 | 8557(14) | 351/8557= 4.1% | 1.02(0.89,1.17) | 0.81(0.69,0.95) |
| 35-39 | 6492(10) | 278/6492= 4.3% | 1.07(0.92,1.24) | 0.86(0.73,1.02) |
| 40-49 | 7954(13) | 427/7954= 5.4% | 1.31(1.14,1.51) | 1.05(0.90,1.23) |
| Malaria |  |  |  |  |
| Yes | 1759(3) | 47/1759= 2.7% | 1.00 | 1.00 |
| No | 60389(97) | 2752/60389= 4.6% | 1.60(1.22,2.09) | 1.29(0.99,1.69) |

*model also adjusted for occupation, religion, survey round and region of residence, EIA_J_ = Enzyme-linked Immunoassay indeterminate
