# Supplementary material for: HIV serologically indeterminate individuals: Future HIV status and risk factors
Source: PLoS One. 2020 Aug 26;15(8):e0237633. doi: 10.1371/journal.pone.0237633 (PMC7449388; doi:10.1371/journal.pone.0237633)
Supplement: Attachment — Submitted filename: Response to reviews 11-26-2019.docx [file pone.0237633.s010.docx]

The Editors, PLOS ONE

Editorial Office

November 26, 2019

Dear Editor,

On behalf of my co-authors, I resubmit the enclosed manuscript under the new title, “HIV serologically indeterminate individuals: future HIV status and risk factors” to be considered for publication in PLOS ONE. We thank the editors and reviewers for their valuable comments and suggestions. We provide an edited and clean version of the manuscript. We believe the present manuscript is greatly improved and we thank you for considering our work for publication.

Below is a point-by-point address of our responses to the reviewers.

Sincerely,

Oliver Laeyendecker

Oliver Laeyendecker MS, MBA, PhD

Staff Scientist, NIAID, NIH

Assistant Professor of Medicine, SOM, JHU

Assistant Professor of Epidemiology, JHSPH

855 North Wolfe St.

Rangos Building, room 538A

Baltimore MD, 21205

Phone: 410-502-3268

Email: [olaeyen1@jhmi.edu](mailto:olaeyen1@jhmi.edu)

Editor’s comment . Please provide additional details regarding participant consent. In the ethics statement in the Methods and online submission information, as your study included minors, state whether you obtained consent from parents or guardians. If the need for consent was waived by the ethics committee, please include this information.

**Response: We thank you for drawing our attention to this important information. The ethics statement has been revised as follows “****All subjects ≥18 years of age provided written informed consent and minors gave assent with parental/guardian consent to participate in the study.”**


Review Comments to the Author

Reviewer #1: Indeterminate test results remain a bane of many within the HIV diagnostics testing arena. While this phenomenon is often accepted as a consequence of testing biological specimens, this manuscript has attempted to quantify the impact of this phenomenon using a statistical lens. While very interesting, this reviewer has concerns re: methodology used from the perspective of the test kits used:

The number (6) and nature of the tests used in the 13 phases is a bit concerning as far as generalizability is concerned. They are not consistent and while sensitivity remains 100% the specificity does not. The Welcozyme EIA used in the later phases has 99 % specificity. Considering the range of specificities reported by the different manufacturers, this parameter would be expected to have a potentially significant impact on indeterminate (false-reactive) test results.

**Response: We thank the reviewer for their comment and are addressing these issues below.**

2. Because of this can the authors stratify their analysis so that the same EIAs used in the parallel testing algorithm are consistent between phases. By admission the authors state that the same pair of EIAs were used in the first 8 phases. Emphasis should be placed on this phase of the test results to avoid generalizing with later phases that employed different EIAs.

**Response: To determine how the change in test kit over time might impact our results, we did a sensitivity analysis limiting the study to round 1 through round 8 where the same pair of test kits were used. The inference did not change when we limited the analysis to these rounds (See supplementary Table 4., Supplemental Digital Content 9). We added the following sentence to the end of the second paragraph of the results section, “When the analysis was limited to the first eight rounds, where the testing was performed with the same pair of kits, the same inferences held (****See supplementary Table 4., Supplemental Digital Content 9).”**

3. The stratification should also distinguish the antigens used between the different EIAs. As an example any EIA employing whole-viral lysate will likely increase false-reactives while those employing synthetic peptides/recombinant antigen would be expected to reduce false-reactives. The discussion should also include this variation between the different EIAs as a limitation to their analysis.

**Response: We thank the reviewer for drawing our attention to this important point. We have added the antigens for each test kits into the supplemental digital content 7, which now shows the test kit, the manufacturers reported sensitivity and specificity and the antigens as well. Due to the nature of the testing we are not able to stratify our analysis by test kit antigens. This will be taken into consideration in future studies when it is possible to stratify by antigens.**

**In addition, we have added the following sentence to the limitations “Test kits that used whole viral lysates are known to have high false positive rate and we were not able to stratify our analysis by the antigens used by each test kit.”**

4. Regarding generalizability the positive predictive value (PPV) of a screening test/algorithm will be higher in a high-prevalence population. It would be helpful if the authors could consider a sensitivity analysis on what the impact would be in lower-prevalence population such as North America.

**Response: Unfortunately, we do not have a low prevalence population to directly compare our results to. That said, as the proportion of false positives would increase for each test in a lower prevalence population, the proportion of indeterminate results from truly in infected individuals would increase. We have added the following sentence to the limitations paragraph in the discussion. “The results presented in this analysis may not hold for other populations, especially those with lower HIV prevalence. In lower prevalence populations, samples with indeterminate results would have a much lower frequency of being true HIV positive.”**

5. Lines 265-269. While the authors make the statement that guidelines are lacking on persistently indeterminate (serology) test results, they should make reference to the fact that molecular-based tests remain a commonly used testing strategy to help resolve these problematic specimens.

**Response: We thank the reviewer for the suggestion. We have added a sentence to the first paragraph of the discussion that states: “To date, molecular-based test such as PCR are commonly used to try to resolve discordant HIV ELISA results, which sometimes can still give inconclusive results. Also, PCR may not be feasible in remote hard to reach settings in low and middle income countries.”**

6. Line 256. Spelling/grammar; determine(d), the authors should do a better spellcheck as the manuscript is peppered with spelling errors.

**Response: We thank the reviewer for their careful reading of the paper and have made corrections to the document.**

7. Line 147. There is an interesting finding re: lower indeterminate result in those residing in urban/trading vs rural residence. Could the authors speculate on why this may be?

**Response: We speculate that people who reside in rural communities in Uganda are mostly farmers who are exposed to tetanus (from farm related injuries) and other infections as a result of their farming activities. Such individuals’ immune systems would be more exposed to a broad array of infections and likely generate more cross-reactive antibodies. This in part may explain why we see high prevalence of indeterminates among people in rural areas compared to people who reside in urban/trading areas.**
