# Supplementary material for: HIV serologically indeterminate individuals: Future HIV status and risk factors
Source: PLoS One. 2020 Aug 26;15(8):e0237633. doi: 10.1371/journal.pone.0237633 (PMC7449388; doi:10.1371/journal.pone.0237633)
Supplement: Attachment — Submitted filename: Rebuttal Letter with responds to reviewers.docx [file pone.0237633.s011.docx]

**Dear Editor,**

**Thank you for the opportunity to revise and resubmit our manuscript, HIV serologically indeterminate individuals: future HIV status and risk factors,” for consideration for publication in the PLOS ONE journal. Please find below a point-by-point response to the reviewers’ comments and corresponding changes made to the manuscript. We have revised the manuscript incorporating nearly all of the reviewer’s recommendations and agree that these revisions have strengthened the quality of the article. Please let us know if we can be of any additional assistance in the review of our manuscript.**

**Thank you again for your consideration.**

Reviewer #1: (No Response)

Reviewer #2: This paper is quite interesting and presents an interesting phenomenon worthy of publication and reflection. Some significant gaps, however, likely first need address.

Major comments
1. This issue of indeterminate results seems focused on EIA and ELISAs; however, both of those test types are very rarely used for clinical management of patients. Instead RDTs are used. It would be helpful to understand and discuss if this might be a phenomenon for RDTs in their current contexts. Further, it wasn’t entirely clear whether the focus and suggested consideration of these results should be primarily or solely in surveillance studies or actual clinical management and diagnosis.

**Response: We thank the reviewer for their thoughtful comment. The reviewer is right, the paper is focused on EIA and ELISAs and not on RDTs. Since the paper did not look at RDTs it is impossible to determine if our findings will be applicable to RDTs. ELISAs are used both for surveillance and clinical diagnoses hence we cannot limit our findings to only clinical or surveillance scenarios. In any situation (surveillance or clinical) where EIA and ELISAs are used in the process, our findings may be applicable. In future we plan to examine indeterminate results for RDTs.**

**We added the following sentence to the manuscript to address this comment “The findings from this study are applicable whenever ELISAs are used either for HIV surveillance or clinical diagnoses purposes.”**

2. It would be very helpful to indicate somewhere (partly in the abstract, but definitely in the methods) the variety of different behavioral, health, demographic and socioeconomic information collected from each patient. Also, whether this was consistently collected across time points.

**Response: We thank the reviewer for drawing our attention to this important point. The following sentence has been modified to capture both the information collected and the idea that this was consistent over all surveys. We provided reference to earlier publications from RCCs which include details on all the behavioral, health, demographic and socioeconomic information collected from each participant.**

**“Behavioral (e.g. number of sexual partners, alcohol use, religion), health (e.g. malaria infection), demographic (e.g. age, sex, marital status) and socioeconomic (e.g. education level, occupation) information as well as blood samples for HIV testing were consistently collected across all surveys(1,2).”**

3. Some of the more important references provided throughout the introduction are incredibly old (+10 years) and should be updated or else the implications lightened. For example, the WHO recommendation is from 1997 and unlikely to still exist and be relevant. This study itself was done between 1994 and 2009, ending over 10 years ago. This absolutely must be included as a limitation and perhaps clearly and specifically discussed with reference to the results – are/can they still be considered valid given the significant changes in policy and the epidemic since then. Further, it’s unclear if the patients included were known HIV-infected and whether on treatment. If not, that changes the implications of the results given the population of HIV-uninfected and untreated is getting increasingly smaller.

**Response: We agree with the reviewer that some of the citations are old and that the data for the study itself is old. However, current information on parallel HIV ELISAs are rare, yet these assays are still used for HIV testing today and the issue of indeterminate results persist. Updated information on HIV ELISA indeterminate results are quite limited in the literature which motivated us to carry out our analysis. We indicated the time period (as correctly reference by the reviewer) when the data for our analysis was collected, the test kits and other details to give readers the opportunity to decide whether our findings will be applicable in their situation.**

**Treatment became available in Rakai Uganda as part of PEPFAR in 2006. For the time period of this study individuals who had a CD4 count <250 cells/mm3 would be eligible for treatment. When we limited the analysis to the first 8 rounds which occurred between 1994 to 2002 when ART was not available in Rakai, the inference did not change. (See supplementary Table 4., Supplemental Digital Content 9). We did include the following sentence in the manuscript to acknowledge that the data used in the analysis is old. “Additionally, the results from this study should be interpreted with caution given the changes in the HIV epidemic and testing tools over the years.”**

4. In the introduction, a number of similar previous works are referenced; however, neither there nor more importantly in the discussion are these discussed and the importance of the current work to that body touched on.

**Response: We thank the reviewer for this comment. In the introduction we did indicate the gaps in the existing studies stating that most of the studies have relatively smaller sample size, short follow-up time for people with indeterminate results making it impossible to understand the long-term outcomes of people with HIV indeterminate results. We provided justifications for why we think the current study is important for the advancement of HIV testing.**

**Throughout the discussion we cited several papers that were initially cited in the introduction section (~8 papers cited in the introduction) to help put our findings in context of the existing literature. For instance, in the discussion we compared the prevalence, correlates and subsequent outcomes of indeterminate results in our study to other studies (citing studies from our introduction). We will be citing additional studies from the studies cited in the introduction to address comment number 5 below.**

5. There were several interesting findings, many of which were identified previously, such as males and those married were at higher risk of indeterminate rates. However, this should be discussed further. Why might these issues be happening in those populations?

**Response: We thank the reviewer for drawing our attention to this important point. We have added the following sentences to further elaborate on our findings:**

**“Married women in Rakai are less likely to use contraceptives and more likely to have children(3,4) and parity has been found to be associated with having indeterminate results among females(5). False HIV EIA positive results has also been reported to be associated with multiparous women(6).”**

**“In Uganda, males have higher prevalence of smoking compared to females(7) and it is well established that cigarette smoking can alter blood viscosity(8). Viscous blood samples or samples with precipitates can form residues which could interfere with ELISA assays(9). This, in part, may explain why males have higher prevalence of indeterminate results compared to females and it is important for public health screening programs to consider this when screening males for HIV infection.”**

6. It would also be helpful to understand if any combinations of the factors may result in more indeterminate results. For example, older married males or need those factors always be disaggregated?

**Response: We thank the reviewer for the idea of exploring if there is interactive effect. The original goal of this study was not to examine the combined effect of any of the factors. We believe the exploration of the combined effect of multiple indicators and their effect on indeterminate results is a completely different research question which is not a part of the goal of our analysis. This will be considered in future studies.**

7. I’m not sure lines 219-238 bring a lot to the results given all others. I might suggest removing, particularly to include a possible analysis per point #6.

**Response: Lines 219-238 referenced by the reviewer presents the results for one of the key objectives of the study i.e. the long-term outcomes of people with initially indeterminate results. Removing these lines in our view will defeat the primary goal of the study.**

8. It would also be helpful to more clearly discuss possible reasons for why indeterminate results may be persistent. What could be causing this? Lines 272-279. Also, what can or should be done for those patients?

**Response:** **We added some more some more explanations that possibly result in persistent indeterminate results. We added the following sentence to further explain why indeterminate results may be persistent:**

**“Some of the factors (systematic lupus erythematosus, rheumatoid factor and polyclonal gammopathy, antibodies to DR-HLA, cross reactivity to core proteins of other retroviruses, mycobacterium leprae infection, in vitro hemolysis, parity and tetanus vaccination(10)) known to be associated with indeterminate results are chronic or permanent which in part may explain why certain people may remain indeterminate for a long period of time.”**

9. It also might be worth reflecting on the large sample size. No sample size calculation was included and though some of the differences are relatively small, the incredibly large sample size would cause significance.

**Response: We thank the reviewer for this comment. The following sentence has been added to the limitations section: “The large sample could also result in statistical significance.”**

Minor comments
1. Line 36: ‘serological’ results rather than ‘serologic’. Also, ‘immunoassays’ is likely the best terminology.

**Response: We thank the reviewer for thoroughly reading our paper. The above error has been fixed.**

2. The suggestion that those with primary education are less likely to have indeterminate results should be more carefully considered given the adjPR crosses 1.

**Response: We thank the reviewer for drawing our attention to this point. We were interpreting the point estimate and we were not necessarily referring to statistical significance in this situation.**

3. Line 58, ‘assay’ should likely be ‘assays’ for both.

**Response: We thank the reviewer for thoroughly reading our paper. The above error has been fixed**.

4. Lines 189-206 should be double-spaced.

**Response: We thank the reviewer for thoroughly reading our paper. The above error has been fixed.**

5. Line 259-260: I’m not sure that’s still the case given significant scale-up of HIV viral load testing and tuberculosis testing, both molecular PCR-based.

**Response: We thank the reviewer for their thoughts on this. It is true that there are some advances in scaling up PCR-based HIV viral load and tuberculosis testing, but this is still limited to large central hospitals or labs at the national level in several low- and middle-income countries. We do not believe PCR-based testing is available particularly at the subnational level in several LMICs.**
